# Autism diagnosis differentiates neurophysiological responses to faces in adults with tuberous sclerosis complex

**DOI:** 10.1186/s11689-015-9129-2

**Published:** 2015-10-07

**Authors:** Charlotte Tye, Teresa Farroni, Ágnes Volein, Evelyne Mercure, Leslie Tucker, Mark H. Johnson, Patrick F. Bolton

**Affiliations:** MRC Social, Genetic and Developmental Psychiatry Centre, Institute of Psychiatry, King’s College London, De Crespigny Park, London, SE5 8AF UK; Child and Adolescent Psychiatry, Institute of Psychiatry, King’s College London, London, UK; Dipartimento di Psicologia dello Sviluppo e della Socializzazione, Università di Padova, Padova, Italy; Centre for Brain and Cognitive Development, Birkbeck, University of London, London, UK; Institute of Cognitive Neuroscience, University College London, London, UK

**Keywords:** Autism spectrum disorder, ERP, Face, Gaze, Tuberous sclerosis complex

## Abstract

**Background:**

Autism spectrum disorder (ASD) is a common and highly heritable neurodevelopmental disorder that is likely to be the outcome of complex aetiological mechanisms. One strategy to provide insight is to study ASD within tuberous sclerosis complex (TSC), a rare disorder with a high incidence of ASD, but for which the genetic cause is determined. Individuals with ASD consistently demonstrate face processing impairments, but these have not been examined in adults with TSC using event-related potentials (ERPs) that are able to capture distinct temporal stages of processing.

**Methods:**

For adults with TSC (*n* = 14), 6 of which had a diagnosis of ASD, and control adults (*n* = 13) passively viewed upright and inverted human faces with direct or averted gaze, with concurrent EEG recording. Amplitude and latency of the P1 and N170 ERPs were measured.

**Results:**

Individuals with TSC + ASD exhibited longer N170 latencies to faces compared to typical adults. Typical adults and adults with TSC-only exhibited longer N170 latency to inverted versus upright faces, whereas individuals with TSC + ASD did not show latency differences according to face orientation. In addition, individuals with TSC + ASD showed increased N170 latency to averted compared to direct gaze, which was not demonstrated in typical adults. A reduced lateralization was shown for the TSC + ASD groups on P1 and N170 amplitude.

**Conclusions:**

The findings suggest that individuals with TSC + ASD may have similar electrophysiological abnormalities to idiopathic ASD and are suggestive of developmental delay. Identifying brain-based markers of ASD that are similar in TSC and idiopathic cases is likely to help elucidate the risk pathways to ASD.

**Electronic supplementary material:**

The online version of this article (doi:10.1186/s11689-015-9129-2) contains supplementary material, which is available to authorized users.

## Background

Autism spectrum disorder (ASD) is a common childhood-onset disorder characterized by social and communication impairments and restricted/repetitive behaviours and interests. Family and twin studies indicate that genetic factors play an important role in the aetiology of ASD, but non-genetic factors are also likely to be relevant in causation [1–3]. Molecular genetic studies have identified a number of copy numbers and rare variants that underlie genetic risk in a substantial minority of cases [4, 5]. Genome-wide association studies indicate that other more common variants likely play a role, but the studies reported to date are underpowered to confidently identify the major variants involved [6, 7]. Nevertheless, the findings to date are helping to identify putative neurobiological processes involved in aetiology, with a major focus on the genes involved in synaptic development and function [8]. Neuroimaging studies have also begun to chart the structural and functional correlates of ASD, but findings have been rather inconsistent, possibly reflecting clinical and underlying genetic heterogeneity. One strategy to address the problem of genetic heterogeneity is to study syndromic model systems of ASD where the genetic cause is constrained. Tuberous sclerosis complex (TSC) is a multi-systemic disorder caused by a mutation in TSC1 on chromosome 9q34 or TSC2 on chromosome 16p13.3. Individuals with mutations in the TSC genes develop a variable and diverse array of physical, cognitive and behavioural manifestations [9]. Remarkable progress has been made with regard to clarifying the molecular biology of TSC, and this has led to extremely promising new treatment approaches [10–12].

Several studies have shown that autistic behaviours are frequently observed [13, 14], and between 30 and 60 % of individuals with TSC meet diagnostic criteria for ASD [9, 15–17]. It is likely, therefore, that there are specific risk mechanisms that determine which individuals develop ASD [18]. To shed light on the gene-brain-behaviour risk pathways, an important approach is to examine brain-based candidate biomarkers. In particular, event-related potentials (ERPs) are able to capture fast-occurring altered cognitive processes in individuals of different ages and abilities and thus are suitable for identifying biomarkers of complex neurodevelopmental disorders [19–23]. In order to identify these risk factors, it is important to demonstrate that syndromic ASD (occurring within a syndrome such as TSC) is the same as idiopathic ASD, by examining a domain that is known to be altered in the latter. A strong candidate biomarker of ASD is impairment in the ability to process information from the face, associated with activity in temporal brain regions. Findings from ERP studies indicate abnormal responses to face stimuli in ASD, notably a delayed ‘face-sensitive’ N170 [24], a reduction or absence of the face inversion effect on the visual P1 and N170 amplitude [24–34] and altered processing of gaze direction on the N170 [21, 35, 36]. Abnormal ERP responses to gaze direction are also observed in infant siblings of children with ASD [37, 38] and are predictive of subsequent ASD diagnosis [37]. In addition, while the N170 is larger in the right hemiscalp compared to the left hemiscalp in typically developing individuals, individuals with autism show an atypical bilateral scalp distribution [24, 29, 36, 39] suggestive of abnormal cortical specialization for faces [27]. Altered processing of faces may contribute to the social impairments characterizing the disorder such as impaired eye contact, joint attention and theory of mind, which suggests it is a key brain-based biomarker of risk for ASD.

Given that previous findings suggest an association between the presence of tubers in the temporal regions associated with face processing and risk of ASD in TSC [40], there is relatively little work examining whether the same neurophysiological correlates of impaired face processing operate in syndromic ASD. A study of young children with TSC reported delayed N290 (the developmental ‘precursor’ to the N170) and reduced lateralization, effects that were particularly pronounced in children with a diagnosis of ASD [41]. There has not, however, been an investigation of electrophysiological responses to changes in face orientation and gaze direction in adults with TSC.

The current study aimed to examine neurophysiological responses to faces and eye gaze in adults with TSC with and without a diagnosis of ASD, compared to typical adult controls, using a paradigm that has been used in several previous studies of ASD [21, 35, 42, 43]. On the basis of previous work, it was hypothesized that neurophysiological responses to faces would differ in TSC + ASD compared to controls. Specifically, we predicted that adults with TSC + ASD would show (1) slowed processing of faces, (2) a reduced effect of face orientation and (3) altered processing of gaze direction.

## Methods

### Participants

Typical control adults (*n* = 13, male *n* = 11) and adults with TSC (*n* = 14, male *n* = 9) were recruited for the study. Participants with TSC underwent a multidisciplinary clinical assessment, and six participants (male *n* = 5) were given a clinical diagnosis of ASD according to DSM-IV criteria. Participants were aged 16–46 years (mean age = 26.85, SD = 7.29); there were no significant differences in age or gender proportions between groups (Table 1). Intellectual ability was measured using different tools depending on the ability level of the participant (Vineland Adaptive Behaviour Scales, Wechsler Intelligence Scale for Children, Wechsler Adult Intelligence Scale, Raven’s matrices, British Picture Vocabulary Scale), and a proxy IQ combining these measures was created for 13 of the TSC participants (data missing on one participant), similar to previous work in TSC [44]. There was a significant difference between TSC-only and ASD groups in intellectual ability measured using this parameter, driven by two ASD participants with severe intellectual disability (ID) (IQ 20–26). Findings were retained when the analysis was repeated without the two severe ASD + ID participants, and the pattern of results remained the same across all ERP parameters (results available on request). There was no significant difference between the TSC-only and TSC + ASD groups in rates of epilepsy at time of testing, but there was a significant difference in the rates of participants currently taking medication for epilepsy whereby all individuals with ASD were taking medication (Table 1). Where possible, epilepsy severity was calculated using the Early Childhood Epilepsy Severity Scale (E-CHESS) used in previous studies of TSC [45, 44] combining information on seizure frequency, type, duration, treatment and response to treatment (due to lack of consistent questioning on status epilepticus, this parameter was not included in the scale). There was no significant difference in epilepsy severity between the TSC groups (Table 1). Despite group differences, IQ was not a significant covariate and therefore was not retained in the analyses (results remained the same when IQ was included as a covariate; see Additional file 1 for analysis of covariance results). The study protocol was approved by the Cambridge Local Research Ethics Committee and the Department of Psychological Sciences Ethical Committee, Birkbeck, University of London.

**Table 1:** Demographic and clinical characteristics

	Controls	TSC	TSC + ASD	
Age (SD)	29.77 (5.67)	25.50 (8.52)	22.33 (6.92)	n.s.d.
Gender (male, %)	85	50	83	n.s.d.
IQ (SD)	All >70	88.29 (13.64)	53.33 (30.82)	*F* = 7.40, *p* = .02
Current epilepsy	n/a	25 %	50 %	n.s.d
Epilepsy severity (SD)	n/a	1.43 (3.77)	3.75 (2.87)	n.s.d.
Epilepsy medication	n/a	25 %	100 %	*x* ^2^ = 7.88, *p* = .01

*Abbreviations*: *n/a* not applicable, *n.s.d.* non-significant difference

### Task

The stimuli were colour images of three female faces with direct or averted gaze (looking right or left). These images were presented either in upright or inverted orientation on a grey background. Faces subtended 15.8° × 10.2° from a viewing distance of 90 cm. Each trial began with the presentation of a fixation stimulus that had a variable inter-trial interval of 800 and 1200 ms to reduce stimulus repetition effects and ensure the child could not predict the onset of the face stimulus. Face stimuli were presented for 500 ms followed by a 500-ms interval without visual stimulus and were aligned vertically so that the eyes appeared at the same height as the fixation stimuli, in order to orient attention towards the eyes. Four hundred eighty trials were presented with randomized presentation. Participants were asked to count the appearances of flags among the fixation stimuli, in order to stimulate participation and attention, and participants were also continually monitored by video recording. This paradigm has been used previously in ASD and infant samples [21, 35, 42].

### EEG recording and processing

Electroencephalography was recorded using a Hydrocel in control adults and the Geodesic Sensor Net in adults with TSC, each with 128 electrodes (Electrical Geodesics Inc., Eugene, OR). The reference electrode was the vertex (Cz in the conventional 10/20 system). The electrical signal was digitized at a 250-Hz sampling rate and amplified with a 0.1- to 100-Hz band-pass filter. The data were analysed offline using Net Station 4.4 analysis software (Electrical Geodesics Inc.). The continuous electroencephalographic signal was segmented to a 1000-ms period and corrected to the 200-ms baseline prior to stimulus onset. The entire trial was excluded if data from more than 12 channels were removed or if the trial contained blinks or other artefacts, and missing data for trials with 12 or fewer bad channels, irrespective of their location, were interpolated. The remaining segments were visually scanned for bad channels and other artefacts. Participants with fewer than 20 good trials in any condition were excluded from further analysis. There were significant differences in the number of accepted trials between the typical controls and the TSC + ASD group across all conditions (see Additional file 2). Average waveforms for each individual participant were calculated and re-referenced to the average.

**Fig. 1:**
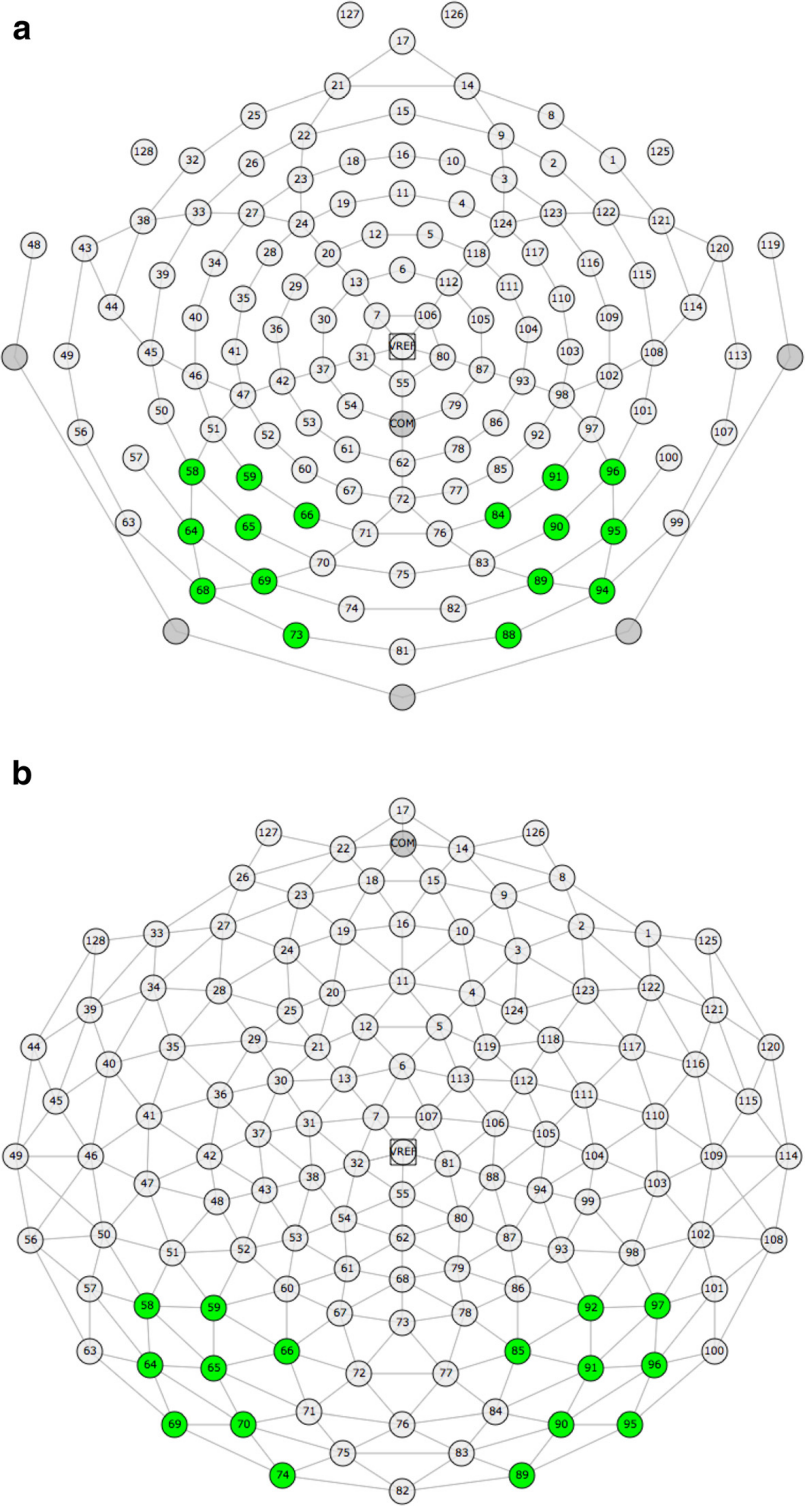
Montages for **a** Geodesic net used with TSC adults and **b** Hydrocel net used with control adults

Based on visual inspection of the grand average and congruent with previous literature, a montage of electrodes was created where the P1 and N170 components were maximal in the right and left occipito-temporal regions, matched over the two EEG recording systems (Geodesic (TSC): left: 58, 59, 64, 65, 66, 69, 70, 74; right: 85, 89, 90, 91, 92, 95, 96, 97; Hydrocel (controls): left: 58, 59, 64, 65, 66, 68, 69, 73; right: 84, 88, 89, 90, 91, 94, 95, 96; see Fig. 1 for montages). Based on visual inspection of the individual data, the latency windows were defined as follows: P1 (74–168 ms), N170 (128–226 ms). A peak-to-peak amplitude was also calculated between the P1 and N170 amplitude. The component peak within this latency window was extracted for each participant, in each condition, for the average of all channels in the left and in the right hemisphere.

### Statistical analysis

A repeated measures ANOVA was conducted on each ERP parameter (P1 amplitude, P1 latency, N170 amplitude, N170 latency) with orientation (upright/inverted), gaze (direct/averted) and hemisphere (left/right) as the within-subjects factors and group as the between-subjects factor (control, TSC-only, TSC + ASD). Age was not significant as a covariate and therefore was dropped from all analyses. Post hoc analyses were carried out when necessary using Fisher’s least significant difference procedure due to the preliminary nature of the analysis. Effect sizes (Cohen’s *d*) were calculated using the difference in the means, divided by the pooled standard deviation of the data.

## Results

**Fig. 2:**
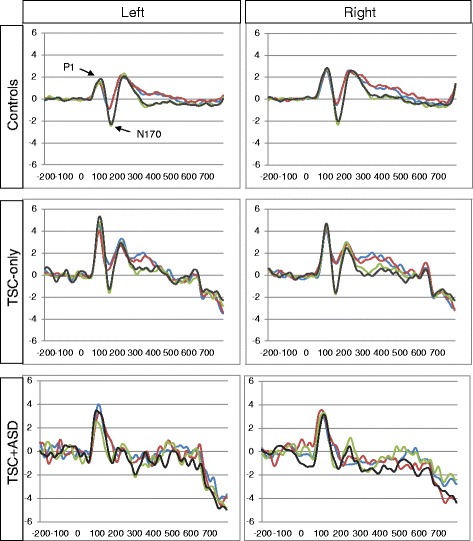
Grand mean ERPs to face stimuli for each group in the left and right hemiscalp. *Blue* represents upright-direct, *red* represents upright-averted, *green* represents inverted-direct, and *black* represents inverted-averted

See Fig. 2 for grand average ERP responses to each stimulus by group. See Additional file 1 for amplitude and latency values for each condition by group and findings of analysis of covariance with IQ to test the significant differences between TSC-only and TSC + ASD.

### P1 amplitude

There was no main effect of age (*F*(1,24) = 1.70, *p* = .21) and age was therefore dropped as a covariate. There was a main effect of group on P1 amplitude (*F*(2,24) = 5.75, *p* = .009). Post hoc analyses showed that controls had reduced P1 amplitude compared to TSC-only (*p* = .006, *d* = 1.36) and TSC + ASD (*p* = .02, *d* = 1.22).

There was no main effect of orientation, but there was an interaction between group and orientation (*F*(2,24) = 6.56, *p* = .005). Significant differences were shown between TSC + ASD and controls (*p* = .03, *d* = 1.33) and between TSC + ASD and TSC-only (*p* = .001, *d* = 1.75). While controls showed little effect of orientation, TSC-only showed greater amplitude to inverted faces whereas TSC + ASD showed greater amplitude to upright faces.

There was an interaction between group and hemisphere (*F*(2,24) = 4.34, *p* = .03). There was a significant difference between controls and TSC-only (*p* = .02, *d* = 0.96) and controls and TSC + ASD (*p* = .02, *d* = 1.49), indicating greater amplitude in the right hemisphere in controls only.

### P1 latency

There was a significant interaction between group and orientation on P1 latency (*F*(2,24) = 7.79, *p* = .002). Post hoc analyses revealed significant differences between TSC + ASD and controls (*p* = .001, *d* = 1.71) and between TSC + ASD and TSC-only (*p* = .03, *d* = 1.15), whereby controls showed longer latency to inverted faces, TSC-only showed a similar response to both upright and inverted faces and TSC + ASD showed a longer latency to upright faces.

There was a significant interaction between gaze and hemisphere (*F*(1,24) = 5.00, *p* = .04) and a three-way interaction between group, gaze and hemisphere (*F*(2,24) = 4.64, *p* = .02). Post hoc analyses indicated significant differences between TSC + ASD and controls (*p* = .03, *d* = 0.92) and between TSC + ASD and TSC-only (*p* = .01, *d* = 1.38); in the left hemisphere, TSC + ASD showed longer latency to averted gaze whereas in the right hemisphere longer latency to direct gaze. Controls and TSC-only showed minimal differences between direct and averted gaze on P1 latency.

### Absolute N170 amplitude

There was a main effect of face orientation (*F*(1,24) = 24.84, *p* > .001), indicating enhanced N170 amplitude to inverted faces compared to upright faces in all three groups. No other effects were shown (all *p* > .05).

### N170 amplitude relative to P1 amplitude

There was a main effect of orientation (*F*(1,24) = 17.06, *p* < .001), indicating enhanced N170 amplitude to inverted faces compared to upright faces. There was a marginally significant interaction between group and hemisphere (*F*(2,24) = 3.07, *p* = .05). Post hoc analyses showed a marginally significant difference between controls and TSC + ASD (*p* = .05, *d* = 1.46), whereby controls showed greater amplitude in the right hemisphere whereas TSC + ASD showed greater amplitude in the left hemisphere. There were no other significant effects (all *p* > .05).

### N170 latency

A number of significant findings were shown for N170 latency. There was a main effect of group (*F*(2,23) = 4.39, *p* = .02), indicating that there was longer N170 latency in TSC + ASD compared to controls (*p* = .01, *d* = 1.46) and a trend for TSC-only (*p* = .09, *d* = 0.96).

There was no significant main effect of orientation (*F*(1,24) = 2.14, *p* = .16) but a significant interaction between group and orientation emerged (*F*(2,24) = 5.75, *p* = .01). Post hoc analyses revealed a significant difference between TSC + ASD and controls (*p* = .002, *d* = 1.56) and between TSC + ASD and TSC-only (*p* = .04, *d* = 0.95), indicating a reduced effect of orientation in TSC + ASD. In addition, a three-way interaction between group, orientation and hemisphere was found (*F*(2,24) = 8.16, *p* = .002). Post hoc analyses on the different scores revealed a significant difference between TSC + ASD and controls (*p* = .001, *d* = 1.54) and between TSC + ASD and TSC-only (*p* = .002, *d* = 1.52). Taken together, these effects showed that controls and TSC-only have a longer N170 latency to inverted compared to direct faces, whereas adults with TSC + ASD showed a reduced effect of orientation particularly in the left hemisphere.

There was a main effect of gaze (*F*(1,24) = 7.02, *p* = .01), and a significant interaction between group and gaze was shown (*F*(2,24) = 4.00, *p* = .03). Post hoc analyses indicated that TSC + ASD had a significantly enhanced effect of gaze on N170 latency compared to controls (*p* = .01, *d* = 1.19). Thus, TSC + ASD showed longer latency to averted gaze compared to direct gaze.

## Discussion

The present study examined neurophysiological responses to faces in individuals with TSC, with and without ASD, and typical individuals. Results indicate that adults with TSC and ASD showed altered processing of faces as indexed by the P1 and N170, which are in line with previous work in idiopathic ASD populations.

Firstly, this group had slower processing of faces overall, consistent with previous work [24, 27]. Notably, the face-sensitive N170 component was only altered in TSC + ASD, an effect that was not driven by low-level visual processing as no latency differences were observed on the P1 component. This supports and extends previous findings of prolonged N290 latency in young children with TSC + ASD [41]. This finding was, however, attenuated when IQ was included as a covariate, which could reflect insufficient power when comparing the TSC-only and TSC + ASD groups, or may indicate prolonged N170 latency is a marker of the comorbidity between ASD + ID in TSC. In addition to abnormalities in speed of processing, both TSC groups showed greater P1 amplitude compared to controls, particularly TSC-only, suggesting enhanced low-level visual processing across stimuli. Hyper-sensitivity to perceptual stimuli is widely reported in ASD [46], and abnormal visual processing has been hypothesized as causative in the social problems apparent in ASD [47]. In addition, altered structural connectivity of visual pathways in the brain has been demonstrated in children with TSC [48]. As the enhanced P1 is demonstrated in both TSC groups, this suggests that the association between TSC and ASD is not necessarily a result of impairment in early sensory processing, supported by intact visual evoked potentials in infants with TSC [49]. Further work is required to examine whether enhanced P1 amplitude directly relates to sensory sensitivity. Evidence for altered cortical specialization of faces in individuals with TSC was indicated for P1 latency and for TSC + ASD only on peak-to-peak analyses of N170 amplitude, as shown by greater amplitude in the left hemisphere or bilaterally in this group. This lack of asymmetry has previously been reported in children and adults with ASD [21, 24] and in children with TSC [41]. Given that bilateral responses to faces are demonstrated in younger children [50], this may reflect delayed development of brain regions occurring early in the pathophysiology of TSC.

Consistent with previous work in ASD [24], typical individuals showed longer P1 and N170 latency to upright compared to inverted faces, whereas individuals with TSC + ASD showed minimal differences or a reversed effect. The lack of sensitivity to face inversion has been used to support theories of ‘weak central coherence’ in ASD, referring to a cognitive bias towards local detail [51], which could be associated with a reliance on features to process faces and/or an impairment in configural face processing. In addition, a lack of sensitivity on the N170 to gaze direction in typical adults is consistent with findings demonstrated using the same paradigm [35]. Importantly, young children with ASD show enhanced activity to direct compared to averted gaze [35], an effect similar to that shown in typically developing infants [43]. In late childhood, sensitivity to averted gaze demonstrated in typical controls is not shown in ASD [21]. The current findings suggest developmental delay of gaze processing in the TSC + ASD group relative to typical adults.

Taken together, the findings demonstrate that individuals with TSC generally show abnormal neural responses to faces at early visual processing stages as indexed by the P1. Importantly, individuals with TSC that also have a diagnosis of ASD demonstrate altered processing of faces as indexed by the face-sensitive N170 component and altered gaze processing as indexed by both the P1 and N170 components, indicative of brain-based biomarkers that are specific to ASD and similar to idiopathic ASD. The results of the study emphasize the importance of using syndromic models of ASD to provide insight on gene-brain-behaviour relationships. Importantly, future work in prospective longitudinal studies will reveal whether specific brain abnormalities or epilepsy-related factors associated with the pathophysiology of TSC are causally linked to altered neurophysiological responses to faces [e.g. 52]. For example, the early arising structural brain changes occurring in TSC may impact the development of brain regions implicated in basic visual processing in TSC, but visual abnormalities might not be directly related to abnormal face processing in ASD within TSC. Examining these indices prospectively will reveal the links between early visual processing and development of the brain network that will later specialize for face processing [53, 54]. If there is a key primary deficit, treatment directed at that risk pathway may be very effective. By contrast, if there are multiple primary deficits, then intervention may need to be targeted at each risk pathway.

The preliminary nature of this work limits power to make firm conclusions but lays the foundation for future replication studies in larger samples. Despite the relative aetiological homogeneity of TSC, it is important to note that individuals with TSC and ASD are likely to have other comorbidities, such as epilepsy and intellectual disability, and therefore a more complex phenotype. We were not able to gather IQ data in the typical adults, rendering it difficult to control for the effect of cognitive ability, although findings were retained when only the high-functioning adults with TSC were included and when IQ was included as a covariate. An ideal future analysis will compare IQ-matched controls with and without a diagnosis of autism to individuals with TSC with and without a diagnosis of autism to ascertain the validity of a phenotypic comparison and support our conclusions. Future research should be designed that enables identification of biomarkers that differ between ASD and ID in TSC. In addition, a significantly higher proportion of individuals with TSC and ASD were taking seizure medication; although this was not a significant covariate, this may confound the findings. Still, the current findings suggest a level of homology between syndromic and non-syndromic autism, which warrants further work to refine the autism phenotype within TSC. Given the dimensional nature of ASD symptoms, future work should consider symptom severity scores and their relationship to candidate biomarkers in addition to a categorical distinction. Neurophysiological indices of other cognitive domains need to be explored in order to support the homology between biomarkers of ASD in TSC and idiopathic ASD.

## Conclusions

This study is the first to characterize candidate electrophysiological biomarkers of face and gaze processing in adults with TSC and ASD. The identification of aberrant neural correlates of face processing that are similar reduces the distinction between syndromic and non-syndromic cases. With additional characterization, neurophysiological profiles of ASD in TSC may serve as valuable biomarkers to ultimately elucidate risk pathways to ASD, in order to direct and monitor specific therapeutic strategies.

## Additional files

Additional file 1:
**Supplementary data.** Group differences between TSC-only and TSC + ASD with IQ as a covariate. Additional file 2: Tables S1–S3. Table S1: Mean (SD) number of segments in each ERP average per stimulus and group during the face and gaze processing task. Table S2: Mean (SD) amplitude (in μV) and latency (in ms) for the P1 for each stimulus by group. Table S3: Mean amplitude (in μV) and latency (in ms) for the N170 for each stimulus by group.
