# Supplementary material for: Autism diagnosis differentiates neurophysiological responses to faces in adults with tuberous sclerosis complex
Source: J Neurodev Disord. 2015 Oct 7;7:33. doi: 10.1186/s11689-015-9129-2 (PMC4597757; doi:10.1186/s11689-015-9129-2)
Supplement: Additional file 1: — Supplementary data. Group differences between TSC-only and TSC + ASD with IQ as a covariate. [file 11689_2015_9129_MOESM1_ESM.pdf]

**Table S1:** Mean (SD) number of segments in each ERP average per stimulus and group during the face and gaze processing task

| Stimulus type | Controls | TSC-only | TSC+ASD | Post-hoc |
|---|---|---|---|---|
| Upright, direct | 104 (12) | 79 (29) | 57 (31) | Control > TSC+ASD |
| Upright, averted | 103 (12) | 78 (30) | 55 (27) | Control > TSC+ASD |
| Inverted, direct | 103 (11) | 82 (31) | 58 (31) | Control > TSC+ASD |
| Inverted, averted | 104 (11) | 78 (34) | 56 (30) | Control > TSC+ASD |

**Table S2: Mean (SD) amplitude (in μV) and latency (in msec) for the P1 for each stimulus by group**

| | **Stimulus** | **Hemi-sphere** | **Controls (n=13)** | **TSC-only (n=8)** | **TSC+ASD (n=6)** |
|---|---|---|---|---|---|
| **Amplitude** | Upright, direct | Left | 2.82 (1.54) | 5.97 (2.26) | 6.30 (2.030 |
| | | Right | 4.09 (2.47) | 5.90 (2.21) | 5.52 (1.42) |
| | Upright, averted | Left | 2.86 (1.64) | 5.59 (2.62) | 6.06 (1.16) |
| | | Right | 4.31 (2.32) | 5.91 (2.75) | 6.43 (2.42) |
| | Inverted, direct | Left | 2.85 (1.63) | 6.32 (2.21) | 5.94 (2.16) |
| | | Right | 3.90 (2.03) | 6.03 (2.27) | 5.66 (1.93) |
| | Inverted, averted | Left | 3.05 (1.74) | 6.60 (2.46) | 5.38 (2.07) |
| | | Right | 4.28 (2.00) | 6.10 (2.57) | 4.92 (2.06) |
| **Latency** | Upright, direct | Left | 103.00 (15.54) | 112.13 (13.54) | 123.25 (17.88) |
| | | Right | 108.69 (16.66) | 116.44 (15.92) | 127.75 (22.74) |
| | Upright, averted | Left | 104.89 (13.81) | 110.56 (13.60) | 128.00 (19.31) |
| | | Right | 106.77 (20.36) | 115.81 (14.35) | 116.67 (15.85) |
| | Inverted, direct | Left | 108.58 (18.87) | 113.75 (11.12) | 107.58 (11.31) |
| | | Right | 112.96 (20.20) | 113.69 (11.69) | 113.75 (14.41) |
| | Inverted, averted | Left | 107.38 (19.38) | 111.44 (10.30) | 118.17 (19.45) |
| | | Right | 112.00 (21.95) | 114.44 (11.00) | 116.42 (22.48) |

**Table S3: Mean amplitude (in μV) and latency (in msec) for the N170 for each stimulus by group**

| | Stimulus | Hemi-sphere | Controls (n=13) | TSC-only (n=8) | TSC+ASD (n=6) |
|---|---|---|---|---|---|
| **Amplitude** | Upright, direct | Left | -1.83 (1.74) | -1.15 (2.91) | -2.64 (3.68) |
| | | Right | -1.53 (1.92) | -1.00 (3.59) | -2.37 (3.50) |
| | Upright, averted | Left | -1.78 (1.74) | -1.82 (2.77) | -3.02 (3.17) |
| | | Right | -1.54 (2.00) | -1.22 (3.60) | -2.54 (3.75) |
| | Inverted, direct | Left | -3.49 (3.01) | -3.12 (4.17) | -3.48 (3.13) |
| | | Right | -3.61 (2.85) | -3.07 (5.59) | -3.59 (3.41) |
| | Inverted, averted | Left | -3.43 (3.01) | -2.88 (4.44) | -3.68 (4.48) |
| | | Right | -3.41 (2.68) | -3.30 (5.61) | -3.93 (3.72) |
| **Latency** | Upright, direct | Left | 157.23 (12.36) | 165.50 (19.18) | 191.33 (20.03) |
| | | Right | 160.96 (17.85) | 174.06 (18.16) | 180.33 (32.36) |
| | Upright, averted | Left | 158.23 (16.80) | 167.38 (27.37) | 195.42 (25.23) |
| | | Right | 160.81 (17.11) | 171.75 (21.13) | 194.50 (24.07) |
| | Inverted, direct | Left | 171.04 (13.04) | 169.00 (19.57) | 171.00 (31.99) |
| | | Right | 170.23 (11.37) | 172.00 (15.88) | 188.50 (25.17) |
| | Inverted, averted | Left | 167.27 (13.73) | 175.81 (17.82) | 180.25 (26.90) |
| | | Right | 168.73 (15.63) | 181.81 (22.66) | 196.50 (23.11) |
