# Supplementary material for: Autism diagnosis differentiates neurophysiological responses to faces in adults with tuberous sclerosis complex
Source: J Neurodev Disord. 2015 Oct 7;7:33. doi: 10.1186/s11689-015-9129-2 (PMC4597757; doi:10.1186/s11689-015-9129-2)
Supplement: Additional file 2: Tables S1–S3. — Table S1: Mean (SD) number of segments in each ERP average per stimulus and group during the face and gaze processing task. Table S2: Mean (SD) amplitude (in μV) and latency (in ms) for the P1 for each stimulus by group. Table S3: Mean amplitude (in μV) and latency (in ms) for the N170 for each stimulus by group. [file 11689_2015_9129_MOESM2_ESM.docx]

Supplementary data

Group differences between TSC-only and TSC+ASD with IQ as a covariate

*P1 amplitude*

The interaction between group and orientation was retained [F(1,10)=11.16, p=.007, d=1.86), whereby TSC-only showed greater amplitude to inverted faces whereas TSC+ASD showed greater amplitude to upright faces.

*P1 latency*

There was a trend toward interaction between group and orientation on P1 latency [F(1,10)=3.74, p=.08, d=1.02), whereby TSC-only showed a similar response to both upright and inverted faces and TSC+ASD showed a longer latency to upright faces.

The three way interaction between group, gaze and hemisphere was retained [F(1,10)=6.33, p=.03, d=1.87]. In the left hemisphere TSC+ASD showed longer latency to averted gaze whereas in the right hemisphere longer latency to direct gaze. TSC-only showed minimal differences between direct and averted gaze on P1 latency.

*N170 latency*

The main effect of group was no longer significant [F(1,10)=1.45, p=.25, d=0.66). The three-way interaction between group, orientation and hemisphere was retained [F(1,10)=6.80, p=.02, d=1.44]. These effects showed that TSC-only have a longer N170 latency to inverted compared to direct faces, whereas adults with TSC+ASD showed a reduced effect of orientation particularly in the left hemisphere.
